# The impact on malaria of biannual treatment with azithromycin in children age less than 5 years: a prospective study

**DOI:** 10.1186/s12936-019-2914-8

**Published:** 2019-08-23

**Authors:** Evan M. Bloch, Beatriz Munoz, Zakayo Mrango, Jerusha Weaver, Leonard E. G. Mboera, Tom M. Lietman, David J. Sullivan, Sheila K. West

**Affiliations:** 1 Department of Pathology, Johns Hopkins School of Medicine, 600 N. Wolfe St/Carnegie 446 D1, Baltimore, MD 21287 USA; 2 Dana Center for Preventive Ophthalmology, Johns Hopkins School of Medicine, Baltimore, MD USA; 3 National Institute for Medical Research, Kilosa, Tanzania; 4 National Institute for Medical Research, Dar es Salaam, Tanzania; 5 Francis I Proctor Foundation, University of California, San Francisco, San Francisco, CA USA; 6 Johns Hopkins Bloomberg School of Public Health, Baltimore, MD USA

**Keywords:** Malaria, Clinical trial, Azithromycin, Child mortality, Tanzania

## Abstract

**Background:**

The MORDOR study, a cluster randomized clinical trial, showed that single-dose azithromycin (20 mg/kg) administered biannually for 2 years to preschool children reduced mortality; a study was conducted to determine its effect on clinical symptomatic episodes of malaria as a potential mechanism for mortality benefit.

**Methods:**

A randomized control trial (RCT) was conducted, whereby 30 randomly selected communities in Kilosa District, Tanzania were randomized to receive 6-monthly treatment of children ages 1–59 months with single-dose azithromycin (20 mg/kg) vs. placebo. A prospective cohort study was nested within the RCT: children, aged 1 to 35 months at baseline, were randomly selected in each community and evaluated at 6-monthly intervals for 2 years. At each visit, the children were assessed for recent or ongoing fever and anti-malarial treatment; a rapid diagnostic test (RDT) for malaria was performed. The two major outcomes of interest were prevalence of RDT positivity and clinical malaria. The latter was defined as RDT-positivity with fever at time of evaluation and/or reported fever in the 3 days prior to evaluation. Methods that account for correlations at community level and within individuals over time were used to evaluate associations.

**Results:**

At baseline, the prevalence rates in the children in the azithromycin and placebo arms were 17.6% vs. 15.5% for RDT positivity (p = 0.76) and 6.1% vs. 4.3% (p = 0.56) for clinical malaria. There was a decline in both RDT-positivity and clinical malaria over time in both arms. The difference by treatment assignment was not significant for clinical malaria; it was significant for RDT-positivity with greater odds of decline in the placebo arm (p = 0.01).

**Conclusions:**

Lack of evidence for a significant difference in the prevalence of clinical malaria in children at any visit following treatment suggests that the effect of single-dose azithromycin on malaria is at best transient and limited in scope. Chance overrepresentation of non-seasonal transmission in the communities in the azithromycin arm may account for higher rates of RDT-positivity and less decline over time.

*Trial registration* Clinicaltrials.gov NCT02047981

## Background

The MORDOR study, a multinational cluster randomized clinical trial found that biannual mass treatment of preschool children with azithromycin, was associated with a reduction in all-cause mortality [1]. The mechanism for protective effect remains unclear. Azithromycin is a broad-spectrum antibiotic, that is effective against a diverse array of respiratory [2] and gastrointestinal pathogens [3]. In particular, azithromycin has also been shown to be effective against protozoal infections: specifically, azithromycin—in combination with atovaquone—is the mainstay of therapy for babesiosis [4, 5]. It has also demonstrated moderate efficacy against malaria in laboratory [6–8] and clinical studies [9–11] alike. While azithromycin monotherapy is not recommended as treatment [11], it may be viable in combination with other anti-malarials (e.g. chloroquine) [9]. Given its favourable safety profile, azithromycin is attractive for the treatment of malaria in children and pregnant women [9]. Nonetheless, longitudinal studies of mass drug administration (MDA) of azithromycin have suggested azithromycin’s protective effect to be evident for only a short time for respiratory [12], diarrhoeal disease [13], and malaria parasitaemia [14], which are the major causes of childhood deaths in low-income countries [15].

In the morbidity component of the MORDOR study, a cohort of children, residing in 30 randomly selected communities in Kilosa, Tanzania, were recruited to determine the effect of azithromycin on morbidity indices that included malaria. Pre-school age children that were living in the selected communities, were randomized to undergo biannual treatment with azithromycin or placebo, were evaluated longitudinally every 6 months for 2 years. The purpose of this study was to determine if there was a preferential decline in malaria in the children residing in the communities in the azithromycin arm.

## Methods

### Overview

The MORDOR trial was a cluster-randomized, placebo-controlled, double-masked clinical trial to evaluate the effect of biannual, single dose azithromycin (20/mg/kg single dose) on mortality in children aged 1–59 months [1]. In Tanzania, the trial was conducted in 644 communities in Kilosa District, Tanzania (January 2015 to August 2017). A morbidity study was embedded into the parent MORDOR trial whereby a random selection of 30 of the 644 participating communities underwent evaluation for morbid outcomes. A cohort study of children ages 1 month to 3 years was nested within the trial whereby participating children were followed every 6 months in these 30 communities, to determine the impact of biannual, single dose azithromycin on malaria over time in this cohort. A cohort study adds power to the study from repeated measures in the same children.

### Setting

Kilosa district is situated in a predominantly rural district in central Tanzania. The climate is tropical to semi-arid with bimodal rainfall: short rains occur October through December and long rains span mid-February through May [16]. The major economic activities in the district include agriculture, raising livestock and small business. The district is considered endemic for malaria [17, 18].

### Eligibility

**Fig. 1:**
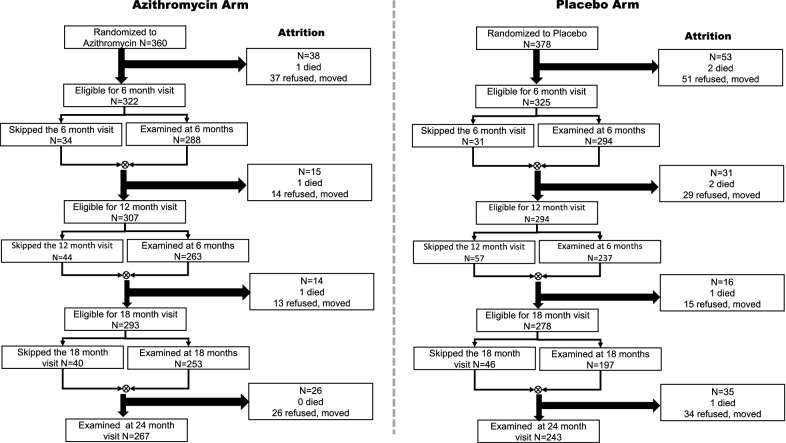
Follow up rates in the cohort over time, by community treatment assignment. Some children missed visits but returned for subsequent visits. The figure reflects those who were also seen in other visits

Thirty communities were randomly assigned to receive biannual treatment of all children aged 1 to 59 months with either azithromycin (20/mg/kg single dose) or placebo. For this morbidity cohort sub-study, children who, at the baseline census, were aged 1 month to 3 years and participated in the baseline survey were enrolled and followed prospectively at 6-monthly intervals for 2 years (Fig. 1). This age group was selected to ensure that no child aged out of the study during the 2 years of follow up.

### Randomization and masking intervention

A randomization sequence was generated using a series of four letters corresponding to the intervention, azithromycin (20 mg/kg) or placebo (Pfizer, New York, NY, USA) with a 1:1 allocation; this assignment was implemented by the Tanzanian study team. The trial was double masked such that the treatment assignment was unknown to the participants and study teams. Only the lead statistician who performed the random assignment was unmasked. The azithromycin and placebo preparations were identical in appearance, presentation and taste.

### Survey

The subjects were evaluated every 6 months, from baseline to 24 months. At each follow-up visit (survey), the guardian was asked if the child had fever in the last 3 days, or was currently taking anti-malaria medication. A rapid diagnostic test (RDT), Paracheck [Orchid Biomedical Systems, Goa, India] for *Plasmodium falciparum* malaria was performed and the results were recorded as positive or negative. Thick blood smears were prepared on the subjects who were RDT positive; these were later stained with Giemsa at a local site laboratory for parasite review. Slide review was performed by a trained microscopist at the local hospital. The child’s temperature was taken to assess for the presence of a fever (defined as axillary temperature ≥ 38 °C). Children who were RDT positive were provided anti-malaria medication (artemisinin-based combination therapy).

### Data analysis

Change in the prevalence of RDT positivity and clinical malaria in the cohort over time were the outcomes of interest. Recent exposure to malaria was defined as positivity on the RDT. Clinically symptomatic malaria was defined as being RDT positive with either the presence of fever OR a report of fever in the 3 days preceding evaluation. Those children who were taking an anti-malarial drug at the time of the survey, were included in a subgroup of clinical malaria.

Bivariate analyses were performed to evaluate cross-sectional differences in malaria indices by treatment assignment, and the Generalized Estimating Equation approach was used to test for differences while accounting for clustering at the community level. The presence of separate outcomes of RDT positivity, and clinically symptomatic malaria were examined for each of the biannual visits. Mixed effect models that include age, time, treatment arm, and the interaction of treatment arm and time as independent predictors were used to evaluate differences between treatment arms in the cohort of children over time. The models account for clustering at community level and repeated measures in the same children over time. At baseline we show the distribution of RDT positivity by community (not at child level) just to show the heterogeneity of the prevalence. This was calculated using the proportion of RDT positivity in the cohort for each community; the results were displayed graphically by treatment arm Analyses were conducted in SAS (Carey NC) using the GEE and GLIMMIX procedures.

### Ethical review and trial oversight

Ethical approval was obtained from the Tanzanian National Institute for Medical Research and the Institutional Review Boards of the Johns Hopkins School of Medicine and University of California San Francisco. Children were included in the study on the basis of documented written informed consent from guardians. The study is registered at clinical trials.gov (NCT02048007). A data and safety monitoring committee provided trial oversight.

## Results

At baseline, there was no significant difference by age, gender, or any of the malaria indices in the children residing in communities randomized to azithromycin or placebo (Table 1). The follow-up rate over the 2 years was 74% in the cohort residing in azithromycin communities and 64% in the placebo communities (cluster adjusted p = 0.08) (Fig. 1). Over the course of the study, there were three deaths in the azithromycin arm, one each after the baseline-, 6-month-, and 12-month visit. There were six deaths in the placebo arm: two after the baseline-, two after the 6-month, one each after the 12-month, and 18-month visits. Both residency in a community that was randomized to placebo treatment, as well as absence of clinical malaria at the previous visit were significant predictors of missing a visit (Table 2).

**Table 1:** Baseline clinical examination results prior to study drug distribution (children 1–36 months at baseline)

Characteristic	Arm AZ, N = 360		Arm PL, N = 378	
	n		n	
Age in months (mean (SD))	360	19.2 (11.0)	378	20.3 (11.0)
% Female	174	48.3	186	49.2
% Child has fever	28	7.8	23	6.1
% Child has had fever in the last 3 days	52	14.5	60	16.0
% Child is taken malaria medication	18	5.0	15	4.0
% Child is RDT positive	63	17.6	58	15.5
% Clinical malaria^a^	22	6.1	16	4.3
% Clinical malaria or taken malaria medication	33	9.2	25	6.7

*AZ* azithromycin, *PL* placebo, *RDT* rapid diagnostic test for malaria, *SD* standard deviation

^a^RDT positive plus current fever or reporting fever in the last 3 days

**Table 2:** Factors associated with children missing visits

Characteristic at the visit 6 months before missing exam	Arm-age adjusted Odds ratio (95% CI), p-value	Multivariate Odds ratio (95% CI), p-value
AZ arm	0.77 (0.61–0.96), 0.02	0.76 (0.60–0.95), 0.017
Age (per month increase)	1.00 (0.99–1.01), 0.86	1.00 (0.99–1.01), 0.81
RDT positive	0.85 (0.63–1.13), 0.26	
Clinical malaria	0.61 (0.38–0.98), 0.04	0.61 (0.38–0.98), 0.04
Taken malaria medication	0.93 (0.49–1.80), 0.84	

*AZ* azithromycin, *RDT* rapid diagnostic test for malaria, *SD* standard deviation

**Fig. 2:**
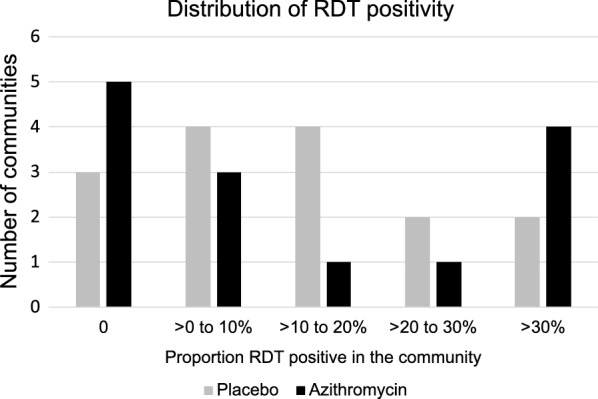
Number of communities with 0 to more than 30% baseline prevalence of RDT positivity in cohort children by treatment arm

At baseline, there was significant variation in the proportion of RDT positive children by community. There were some communities in both arms that had zero prevalence, while two communities in the placebo- and four communities in the azithromycin arm had > 30% prevalence of RDT positivity (Fig. 2).

RDT positivity in the cohort of children showed a seasonal decline at 6- and 18-months for those in the placebo arm; a comparable decline was not shown in the same periods in the children in the azithromycin arm (Table 3). Of all RDT positive cases, 46.9% in the azithromycin and 34.5% in the placebo arm were positive by microscopy.

**Table 3:** Proportion of children with RDT positivity and clinical malaria at each visit by randomization arm

Outcome	Arm	Baseline		6 months		12 months		18 months		24 months		Overall p-value^#^
		N	n (%)	N	n (%)	N	n (%)	N	n (%)	N	n (%)	
RDT+	Placebo	375	58 (15.5)	294	27 (9.8)	237	44 (18.6)	232	10 (4.3)	243	16 (6.6)	< 0.01
	Azithromycin	359	63 (17.6)	288	55 (19.3)	263	57 (21.7)	253	50 (19.8)	266	35 (13.2)	
	p-value*	0.76		0.19		0.66		0.046		0.34		
Clinical malaria	Placebo	375	16 (4.3)	294	8 (2.7)	237	16 (6.8)	232	4 (1.7)	243	3 (1.2)	0.16
	Azithromycin	359	22 (6.1)	288	15 (5.2)	263	16 (6.1)	253	6 (2.4)	266	5 (1.9)	
	p-value*	0.56		0.29		0.83		0.61		0.66		

*RDT* rapid diagnostic test for malaria

*Test for cross-sectional association using Generalized Estimating Equation approach to test for differences while accounting for clustering at the community level

^#^Test for differences across the five visits using test for repeated measures

Overall, accounting for repeated measures over time, there were higher rates of RDT positivity over time in children residing in the communities randomized to azithromycin compared to children residing in the placebo-treated communities (Table 3). However, there was no significant difference in prevalence of clinical malaria at all phases of follow-up.

Adjusting for age as well as the interaction of treatment arm and time, the children residing in the placebo-treated communities showed a modest but significant decline in both RDT positivity and clinical malaria over time (Table 4). The children residing in the azithromycin-treated communities also had a decline in clinical malaria, but not in RDT positivity over time. The difference in the odds of decline in RDT positivity between those children in the placebo vs. the azithromycin communities was statistically significant, p = 0.01.

**Table 4:** Change in outcomes in children by treatment group over time

Outcome	Effect of time (6 months unit) Odds ratio (95% confidence interval)		p-value*
	Azithromycin	Placebo	
RDT positive	0.96 (0.86–1.05)	0.78 (0.68–0.88)	0.01
Clinical malaria	0.76 (0.63–0.92)	0.80 (0.65–1.00)	0.75
Clinical malaria or taken malaria medication	0.74 (0.63–0.88)	0.75 (0.61–0.91)	0.99

*RDT* rapid diagnostic test for malaria

*From mixed effect models that included age at baseline and the interaction between arm and time; p-value for the interaction of arm and time is presented

**Fig. 3:**
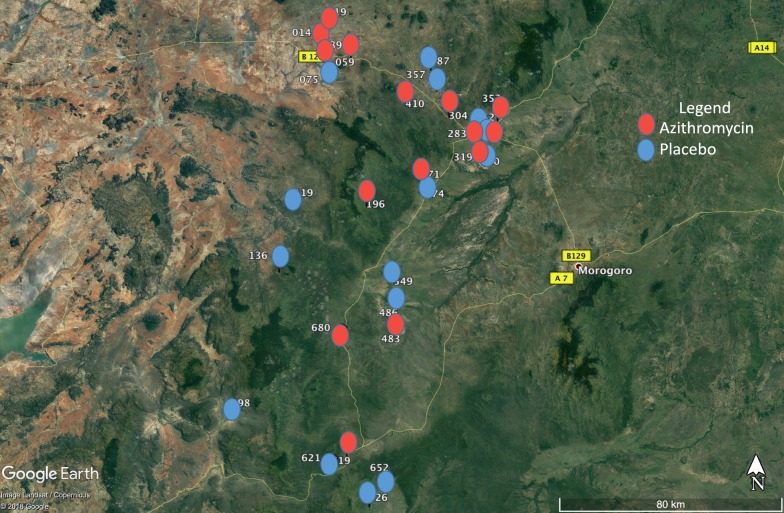
Map of participating communities by treatment assignment

There was no difference in the months or seasons that the surveys were conducted between azithromycin vs. placebo communities at each 6-month interval. A map of the location suggested clustering of intervention villages in north-east Kilosa where malaria was uncommon, and to be in the hills where non-seasonal (i.e. year-round) malaria transmission is more common (Fig. 3).

## Discussion

Globally, malaria remains the foremost parasitic infection in humans. In 2016 alone, there were an estimated 216 million clinical cases of malaria and 445,000 deaths. The overwhelming majority of clinical cases (85%) and malaria deaths (90%) occur in sub-Saharan Africa, of which children under five are disproportionately affected [19]. Despite decades of malaria intervention, over half the world’s population remains at risk, exacting a formidable economic toll in the world’s poorest countries [20]. Germane to Tanzania, there have been significant gains in malaria control as evidenced by a 70% decline in annual cases of malaria from 2008 to 2017. At least in part, this contributed to a contemporaneous 40% reduction in under five mortality, but there are still an estimated 5.5 million cases of malaria, annually, in Tanzania [21].

In a cohort of preschool children in Tanzania, who had been randomized to biannual treatment with azithromycin or placebo, a difference in clinically symptomatic malaria was not shown by treatment arm, 6 months following treatment over a period of 2 years. There was wide variability in the prevalence of RDT positivity across the communities, suggesting absent transmission in some communities yet high rates of transmission in others. Variations in malaria prevalence in Kilosa district has been reported, previously [18, 22]. This heterogeneity is not surprising given the complex interplay of variables that impact transmission in any one location, spanning geography, microclimate, land use, extant mitigation strategies and the myriad of potential breeding sites [16, 18, 22]. There was significant decline in RDT positivity over time in the cohort of children residing in the communities in the placebo group. While there was a decline in the proportion of RDT positive children residing in the communities in the azithromycin arm, it did not attain statistical significance. Overall, there was a comparatively higher proportion of RDT positive children in communities in the azithromycin arm, which suggests that despite randomization, chance overrepresentation of communities with greater, non-seasonal transmission occurred in the azithromycin arm.

Only a small proportion of those who were RDT positive had symptoms (i.e. fever). While RDTs are specific, they fail to discriminate recent infection with absent parasitaemia, from active parasitaemia. This can be challenging in areas of high endemicity, particularly given that *P. falciparum* histidine-rich protein may persist for weeks following treatment [23]. Pertinent to this study, despite lower prevalence of RDT positivity in the placebo group at a community level, the rates of clinical malaria were similar in the children in the azithromycin and placebo arms. One possible explanation is that clinical malaria may be seasonal but rates of RDT positivity are higher in areas with non-seasonal or sustained transmission. As such, despite randomization, the higher number of hyperendemic communities in the azithromycin arm of the study may be masking seasonal fluctuation in the other communities in that arm, and lead to a different pattern as compared to the placebo arm. With only 15 communities in each arm, one cannot definitively determine if this is the case. With respect to symptoms, a comparable rate of fever despite higher rates of RDT positives raises the question as to whether azithromycin may be impacting clinical penetrance i.e. malaria parasitaemia.

A decline in clinical malaria was demonstrated in the children in both arms of the study, which was notable at the last visit. While this may reflect aging of the cohort, it could also reflect the previous year’s active district programme in distribution of insecticide-treated bed nets to pregnant women and children through antenatal clinics (personal communication, Dr. Matthew Lynch, Johns Hopkins Bloomberg School of Public Health, 26 September 2018). Data were not collected on bed net coverage or use, but the decline in RDT positivity and clinical malaria suggest this may be a factor. A Kilosa-based study in 2011 of malaria preventive measures in pregnancy showed that almost all (98%) women of reproductive age had an insecticide-treated bed net in their households [24], suggesting high rates of use once nets are acquired.

Unlike RDT positivity, a significant difference was not found in the rates of clinical malaria in children by community treatment assignment at each 6-month survey after treatment. As described above, despite the higher rates of RDT positivity in the children in the azithromycin arm, it may be that the azithromycin resulted in a lower rate of symptomatic malaria. A previous study found a 66% reduction in the odds of PCR determined infection following mass drug administration for trachom [14]. Nonetheless, the differential effect on PCR evidence of parasitaemia was restricted to the 1st month, with no significant benefit observed at 3, 4- and 6-months following a single treatment [14]. These data suggest it is unlikely that azithromycin had a sustained effect out to 6 months post administration. Similarly, in a clinical trial in Burkina Faso and Mali to evaluate the effect of the addition of azithromycin to a seasonal malaria chemoprevention regimen in preschool children, the prevalence of malaria parasitemia was similar in the azithromycin and placebo groups [25].

Mass drug administration with azithromycin for trachoma control has shown benefit elsewhere. One study in Gambia, evaluated malaria indices in children aged 5–14 years following MDA of azithromycin for trachoma [10]. Four villages were randomly selected to undergo community wide (i.e. all residents) MDA with three doses of azithromycin. Four control villages underwent 6 weeks of daily topical tetracycline. At day 28 following treatment, there was a significant reduction in *P. falciparum*, *Plasmodium malariae*, febrile parasitaemia and rates of palpable splenomegaly in those who received the three doses of azithromycin. Similarly, a cluster randomized trial in Niger showed significantly lower rates of parasitaemia in those communities that underwent two vs. single mass distribution of azithromycin 4–5 months post-treatment [26].

The study has limitations. Foremost, the parent study was conceived primarily to test effect of mass treatment with azithromycin on all-cause mortality. In the event that a benefit was found, the morbidity arm was intended to offer insight into the mechanism for such an effect, specifically on bacterial infections. As such, while the morbidity arm allowed for ancillary study of malaria, rigorous microscopic review was not performed. Microscopy is ideally performed by two skilled microscopists, with a third microscopist used to adjudicate discordant results. Instead, microscopy for this study was undertaken by a clinical technologist at the local hospital in Kilosa. Given resource constraints coupled with the primary scope of the morbidity study, review was restricted to RDT positive cases alone. As such, the findings of a relatively modest rate of smear positivity were not surprising and highlight variable sensitivity of microscopy, which is an observation that has been well documented by others [27–29].

In this regard, RDTs have been pivotal to advancing control strategies for malaria [28]. Their low technical complexity and ease of use with minimal training allows for deployment to low-resource and/or remote areas. This facilitates surveillance and timely referral for treatment optimizing outcomes. RDTs have enabled a shift away from reliance on presumptive diagnosis of malaria based on symptomatology alone.

Second, even with randomization of communities into the placebo and azithromycin arm, chance selection of more villages with year around transmission in the azithromycin arm complicated interpretation of the RDT/clinical symptom results. Even though the district is endemic for malaria, there is clearly significant heterogeneity across and even within communities. This was reported previously, where significant variation in the prevalence of malaria was found at sub village level in Kilosa [16].

Other limitations include some attrition, which resulted in incomplete data on the cohort. However, those with clinical malaria were noted to be more likely to attend the next study visit; this could be attributed to provision of treatment to clinical cases at the time of the survey. Another is the follow-up interval, 6 months after treatment, which is acknowledged to be long in terms of observing an immediate benefit. However, it is uncertain as to whether there might be a longer-term benefit of treating twice a year for 2 years, such that there would be cumulative differences apparent at 2 years. This did not seem to be the case, as seen over time in the cohort analysed by treatment arm at each time point. Finally, there are challenges around the definitions of clinical malaria that were used for this study: for one, fever is non-specific, and it is possible that children were RDT positive and had fever due to another cause, but which was ascribed to malaria. However, if this were the case, it would affect both arms of the study, and should not bias the findings; it would only increase the number of clinical cases. In addition, RDT testing can be negative in those who are already on anti-malarials at time of evaluation. In such cases, the level of antigenaemia may have fallen below the RDT detection limit leading to a false negative despite having recent exposure to malaria. For this reason, this group was included under an alternative definition of clinical malaria and still found no differences.

## Conclusion

While previous research suggests that azithromycin has modest anti-malarial activity, a significant effect on clinical malaria or prevalence of RDT positivity was not demonstrated in these cohorts. Further, no evidence was found to support a reduction in risk of RDT positivity or clinical symptoms over time following multiple 6-monthly azithromycin dosing.

## Data Availability

The datasets used and/or analysed during the current study are available from the corresponding author on reasonable request.

## References

[1] Keenan JD, Bailey RL, West SK, Arzika AM, Hart J, Weaver J (2018). Azithromycin to reduce childhood mortality in sub-Saharan Africa. N Engl J Med.

[2] Gray GC, McPhate DC, Leinonen M, Cassell GH, Deperalta EP, Putnam SD (1998). Weekly oral azithromycin as prophylaxis for agents causing acute respiratory disease. Clin Infect Dis.

[3] Fry AM, Jha HC, Lietman TM, Chaudhary JS, Bhatta RC, Elliott J (2002). Adverse and beneficial secondary effects of mass treatment with azithromycin to eliminate blindness due to trachoma in Nepal. Clin Infect Dis.

[4] Vannier E, Krause PJ (2012). Human babesiosis. N Engl J Med.

[5] Krause PJ, Lepore T, Sikand VK, Gadbaw J, Burke G, Telford SR (2000). Atovaquone and azithromycin for the treatment of babesiosis. N Engl J Med.

[6] Dahl EL, Rosenthal PJ (2007). Multiple antibiotics exert delayed effects against the *Plasmodium falciparum* apicoplast. Antimicrob Agents Chemother.

[7] Sidhu AB, Sun Q, Nkrumah LJ, Dunne MW, Sacchettini JC, Fidock DA (2007). In vitro efficacy, resistance selection, and structural modeling studies implicate the malarial parasite apicoplast as the target of azithromycin. J Biol Chem.

[8] Shimizu S, Osada Y, Kanazawa T, Tanaka Y, Arai M (2010). Suppressive effect of azithromycin on *Plasmodium berghei* mosquito stage development and apicoplast replication. Malar J.

[9] Dunne MW, Singh N, Shukla M, Valecha N, Bhattacharyya PC, Dev V (2005). A multicenter study of azithromycin, alone and in combination with chloroquine, for the treatment of acute uncomplicated *Plasmodium falciparum* malaria in India. J Infect Dis.

[10] Sadiq ST, Glasgow KW, Drakeley CJ, Muller O, Greenwood BM, Mabey DC (1995). Effects of azithromycin on malariometric indices in The Gambia. Lancet.

[11] van Eijk AM, Terlouw DJ. Azithromycin for treating uncomplicated malaria. Cochrane Database Syst Rev. 2011:CD006688. 10.1002/14651858.CD006688.pub2 PMC6532599 21328286

[12] Coles CL, Levens J, Seidman JC, Mkocha H, Munoz B, West S (2012). Mass distribution of azithromycin for trachoma control is associated with short-term reduction in risk of acute lower respiratory infection in young children. Pediatr Infect Dis J.

[13] Coles CL, Seidman JC, Levens J, Mkocha H, Munoz B, West S (2011). Association of mass treatment with azithromycin in trachoma-endemic communities with short-term reduced risk of diarrhea in young children. Am J Trop Med Hyg.

[14] Schachterle SE, Mtove G, Levens JP, Clemens E, Shi L, Raj A (2014). Short-term malaria reduction by single-dose azithromycin during mass drug administration for trachoma, Tanzania. Emerg Infect Dis.

[15] Walker CLF, Rudan I, Liu L, Nair H, Theodoratou E, Bhutta ZA (2013). Global burden of childhood pneumonia and diarrhoea. Lancet.

[16] Paul P, Kangalawe RYM, Mboera LEG (2018). Land-use patterns and their implication on malaria transmission in Kilosa District, Tanzania. Trop Dis Travel Med Vaccines.

[17] Mazigo HD, Rumisha SF, Chiduo MG, Bwana VM, Mboera LEG (2017). Malaria among rice farming communities in Kilangali village, Kilosa district, Central Tanzania: prevalence, intensity and associated factors. Infect Dis Poverty.

[18] Mboera LE, Bwana VM, Rumisha SF, Malima RC, Mlozi MR, Mayala BK (2015). Malaria, anaemia and nutritional status among schoolchildren in relation to ecosystems, livelihoods and health systems in Kilosa District in central Tanzania. BMC Public Health.

[19] White NJ, Pukrittayakamee S, Hien TT, Faiz MA, Mokuolu OA, Dondorp AM (2014). Malaria. Lancet.

[20] Malaria's Impact Worldwide. 2019. https://www.cdc.gov/malaria/malaria_worldwide/impact.html. Accessed 21 Aug 2019.

[21] WHO recognizes national efforts towards malaria elimination. 2018. https://afro.who.int/news/who-recognizes-national-efforts-towards-malaria-elimination. Accessed 21 Aug 2019.

[22] Mboera LE, Bwana VM, Rumisha SF, Stanley G, Tungu PK, Malima RC (2015). Spatial abundance and human biting rate of *Anopheles arabiensis* and *Anopheles funestus* in savannah and rice agro-ecosystems of Central Tanzania. Geospat Health.

[23] Dalrymple U, Arambepola R, Gething PW, Cameron E (2018). How long do rapid diagnostic tests remain positive after anti-malarial treatment?. Malar J.

[24] Rumisha SF, Zinga MM, Fahey CA, Wei D, Bwana VM, Mlozi MR (2014). Accessibility, availability and utilisation of malaria interventions among women of reproductive age in Kilosa district in central Tanzania. BMC Health Serv Res.

[25] Chandramohan D, Dicko A, Zongo I, Sagara I, Cairns M, Kuepfer I (2019). Effect of adding azithromycin to seasonal malaria chemoprevention. N Engl J Med.

[26] Gaynor BD, Amza A, Kadri B, Nassirou B, Lawan O, Maman L (2014). Impact of mass azithromycin distribution on malaria parasitemia during the low-transmission season in Niger: a cluster-randomized trial. Am J Trop Med Hyg.

[27] Wangai LN, Karau MG, Njiruh PN, Sabah O, Kimani FT, Magoma G (2011). Sensitivity of microscopy compared to molecular diagnosis of *P. falciparum*: implications on malaria treatment in epidemic areas in Kenya. Afr J Infect Dis.

[28] Wongsrichanalai C, Barcus MJ, Muth S, Sutamihardja A, Wernsdorfer WH (2007). A review of malaria diagnostic tools: microscopy and rapid diagnostic test (RDT). Am J Trop Med Hyg.

[29] Kilian AH, Metzger WG, Mutschelknauss EJ, Kabagambe G, Langi P, Korte R (2000). Reliability of malaria microscopy in epidemiological studies: results of quality control. Trop Med Int Health.

